# Plasmonic-Enhanced Infrared Absorption Platform for Broadband and Multiple Molecular Fingerprint Retrieval

**DOI:** 10.3390/nano15040284

**Published:** 2025-02-13

**Authors:** Yulong Hu, Zexing Zheng, Huishan Ma, Shuguang Zhu, Yiming Yu, Jie Hong, Weiwei Tang, Jiale He, Libo Zhang, Changlong Liu, Guanhai Li, Xiaoshuang Chen

**Affiliations:** 1 College of Physics and Optoelectronic Engineering, Hangzhou Institute for Advanced Study, University of Chinese Academy of Sciences, Hangzhou 310024, China; huyulong22@mails.ucas.ac.cn (Y.H.); zhengzexing23@mails.ucas.ac.cn (Z.Z.); mahuishan24@mails.ucas.ac.cn (H.M.); zhushuguang23@mails.ucas.ac.cn (S.Z.); yuyiming22@mails.ucas.ac.cn (Y.Y.); hongjie22@mails.ucas.ac.cn (J.H.); kaisafalehua@ucas.ac.cn (J.H.); zhanglibo@ucas.ac.cn (L.Z.); liuchanglong@ucas.ac.cn (C.L.); ghli0120@mail.sitp.ac.cn (G.L.); 2 State Key Laboratory of Infrared Physics, Shanghai Institute of Technical Physics, Chinese Academy of Sciences, Shanghai 200083, China

**Keywords:** mid-infrared, molecular fingerprint, tunable plasmonic metasurface, surface-enhanced infrared absorption

## Abstract

The mid-infrared (mid-IR) region, often referred to as the molecular fingerprint region, encompasses the distinctive absorption spectra characteristic of numerous important molecules. However, the intrinsically small molecular absorption cross-sections, combined with the size mismatch between nanoscale molecules and microscale mid-IR wavelengths, result in inherently weak light-molecule interactions. In this work, we propose a broadband, tunable platform based on plasmonic-enhanced infrared absorption for label-free retrieval of molecular fingerprints. By leveraging the strong near-field enhancement of the plasmonic structure, the platform significantly amplifies light-molecule interactions, enabling precise reconstruction of the fingerprint absorption spectra of target molecules. In addition, the proposed structure exhibits exceptional molecular detection capabilities across the wavelength range of 5–10 μm, with remarkable potential for distinguishing molecular mixture components. The results pave the way for the applications in chemical identification, biomedical diagnostics, environmental monitoring, and other interdisciplinary fields, which require miniaturized and high-precision sensing.

## 1. Introduction

The mid-infrared (mid-IR) spectrum encompasses numerous intrinsic absorption peaks of molecules and atoms, making it one of the most widely utilized spectroscopic techniques. It carries rich vibrational information related to molecular chemical bonds, molecular compositions, and material structures, which makes it indispensable for material identification and structural analysis [1]. However, the small absorption cross-section of individual molecules (approximately 10^−20^ cm^2^ per molecule in the mid-IR region) often limits the sensitivity of trace molecular detection. Moreover, the size mismatch between nanoscale molecular dimensions and the microscale mid-infrared wavelength significantly weakens the interaction between molecules and incident light [2,3,4]. This inherent limitation of weak light–matter interactions poses challenges for molecular fingerprint spectrum detection, leading to large and cumbersome equipment, as well as high detection costs.

Nanomaterials can overcome this limitation by exploiting the strong near-field enhancement of the subwavelength resonator. When the resonance is spectrally overlapped with the absorption fingerprint of the molecules, the interaction between the molecules and the light can be strongly enhanced, and the fingerprint of the molecules can be extracted. From that, it is mainly based on the following two platforms. The first one is based on quasi-bound states in the continuum (quasi-BIC), which are derived from BIC in all-dielectric materials. These sensors capitalize on the theoretically infinite Q-factor of BIC, as well as the strong, zero-leakage interactions it facilitates [5]. The other is based on surface plasmon resonance in noble metal nanostructures, where the surface plasmons (SP) are excited at the interface of the metal and dielectric material, which plays an important role in sensing with high sensitivity. The high sensitivity stems from the local electromagnetic field enhancement and the ultra-sensitivity of the surface plasmon resonance (SPR) to the surrounding medium. For instance, surface-enhanced Raman scattering (SERS) and surface-enhanced infrared absorption (SEIRA) have been realized using a plasmonic platform. In SERS, the enhancement is ascribed to the dominant electromagnetic field contribution due to the excitation of the SPR and the minor chemical contribution originating from the charge transfer effect [6,7]. It is sensitive only to molecules within a few nanometers of the metallic hot spots since the platform is mainly based on a roughened metallic surface. In comparison to its Raman counterpart, SEIRA, based on the resonant nanoantenna, can control the light on the nanoscale and provide a powerful platform to tailor the spectral response and light localization [8,9]. The absorption enhancement of the IR nanoantenna can extend tens of nanometers from its surface by carefully engineering the nanoantenna array, making it suitable for analyzing multilayer assays, which are not assessable by SERS. The implementation of the dielectric metamaterials can realize a high-Q resonance and avoid the Ohmic loss imposed by the plasmonic nanostructures. However, it needs a pixel metamaterial to produce a large number of resonances, making the fabrication difficult [10]. Surface plasmon-based materials typically exhibit lower Q-factors compared to dielectric metamaterials. Nonetheless, they enable the reconstruction of molecular fingerprint spectra within a single periodic structure. For example, Rodrigo et al. (2018) [11] introduced an innovative method that employs self-similar overlapping nanoantenna arrays to construct resonant metamaterials. This design facilitates the generation of two resonant peaks with high absorption, enabling metasurface sensors to simultaneously detect and reconstruct specific molecular fingerprint vibrations. However, for the quasi-BIC-based sensing platform, fingerprint retrieval could be realized by the pixel metasurface, in which each metasurface provides a single narrowband spectrum; hence, a large number of the pixeled metasurfaces with an independent resonant spectrum and a small resonant wavelength gap are needed, which increases the fabrication process with accurate size control among different pixels. For the SPR-based sensing platform, the sensing is limited to distinguishing a single substance related to the structure design, hindering its dynamic range in sensing application. Furthermore, in the mid-infrared region, approaches capable of achieving broad-range and multiple molecular fingerprint detection in a single device remain underdeveloped.

In this work, we present a chemically specific, label-free nanophotonic biosensor for distinguishing multiple analytes with a fingerprint range of 5–10 μm. The sensor uses a simple resonant metasurface constituted by the metal-insulator-metal (MIM) structure. The insulating layer is made of the dynamic material Ge_2_Sb_2_Te_5_ (GST). The device achieves ultra-broadband resonance coverage across the 5–10 μm range by tuning the crystallization degree of the Ge_2_Sb_2_Te_5_ (GST) dielectric spacer layer. Using temporal coupled mode theory (TCMT), we analyze the platform’s absorption and radiation losses to determine its coupling states and assess its ability to reconstruct molecular fingerprint absorption spectra under different coupling states. Furthermore, we exploit this metasurface sensor concept to unravel the interaction of light with different analytes, including polymethyl methacrylate (PMMA), dimethyl methylphosphonate (DMMP), and nitrobenzene (NB) molecules. The findings indicate that the platform can realize fingerprint retrieval with a large wavelength range in a single structure, benefiting from its tunability and broadband properties. In addition, the platform exhibits the ability to determine the mass fraction of the analyte mixtures. Extending this concept to the biomedical field, we can leverage this method to monitor the synaptic vesicle mimics, paving the way for applications in neurobiology and drug development.

## 2. Structural Design and Methods

### 2.1. Structural Design

**Figure 1:**
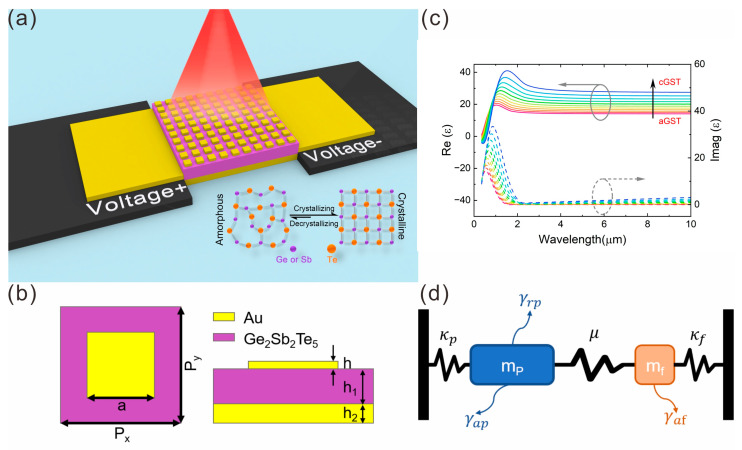
(**a**) Conceptual schematic of a tunable plasmonic-enhanced infrared absorption platform. The inset illustrates molecular diagrams of crystalline GST and amorphous GST materials. (**b**) Structural parameters of a single unit cell: P_x_ = P_y_ = 800 nm, h = 40 nm, a = 450 nm, h_1_ = 180 nm, h_2_ = 100 nm. (**c**) Real and imaginary components of the complex dielectric constant of GST materials at varying crystallization levels, the gradient curves from red to blue represent the transition from amorphous state to crystalline state. (**d**) Conceptual diagram of coupled resonators.

Figure 1a illustrates the structural diagram of the proposed tunable plasmonic-enhanced absorption platform. The central region consists of gold nanostructures, a dielectric spacer layer (GST), and a gold reflection plane. The electrodes on the two sides are used to generate the Ohmic heating to control the degree of the crystallization of GST. Mid-infrared light is incident vertically onto the metasurface. The specific structure of a single-unit cell is shown in Figure 1b. The parameters are as follows: the period P_x_ = P_y_ = 800 nm, the height of the gold nanostructures h = 40 nm, the side length a = 450 nm, the thickness of dielectric spacer layer (GST) h_1_ = 180 nm, and the thickness of the gold film h_2_ = 100 nm. The parameters are obtained by solving the equation of surface plasmon polariton (SPP) excitation conditions of $\left|\vec{k_{SPP}}\right|=\vec{k_{in}}$+$i\vec{G_{x}}$+$j\vec{G_{y}}$, where $\vec{k_{in}}$ is the in-plane component of the wave vector of the incident light with $\vec{k_{in}}=0$ for the normal incidence. The integers i and j denote the resonance order. $\vec{G_{x}}$ and $\vec{G_{y}}$ are the reciprocal lattice vectors with $\vec{G_{x}}=\vec{G_{y}}=\frac{2\pi }{p}$ for the square lattice. The dielectric spacer layer is composed of germanium–antimony–tellurium (Ge_2_Sb_2_Te_5_, commonly referred to as GST), a phase-change material capable of undergoing reversible transitions between crystalline and amorphous states in response to external electric fields [12,13], temperature variations, or optical excitation [12]. Recent experimental studies have further demonstrated that GST can be controllably crystallized into intermediate states [13,14]. A molecular schematic of this phase-change material is provided in the inset of Figure 1a. The thickness of the GST dielectric spacer layer is selected as h_1_ = 180 nm, which maximizes the near-field plasmon coupling strength between the gold nanostructures and continuous gold film. At this thickness, the device achieves near-unity absorption (>99%) due to the fact that the critical coupling conditions are met. The electric field concentrates at the interface between the gold nanostructure and GST, amplifying light–molecule interactions for sensitive fingerprint retrieval.

### 2.2. Materials and Methods

The Finite Difference Time Domain (FDTD) method was employed to numerically simulate the platform. In the calculations, periodic boundary conditions were applied in both the x- and y-directions, while perfectly matched layers (PML) were used along the direction of light propagation (z-axis). The spatial mesh cell is ∆x = ∆y = ∆z = 5 nm, allowing sufficient mesh cells to resolve the layer. The convergence criteria are governed by the Courant–Friedrichs–Lewy (CFL) condition, ensuring that the numerical solution remains stable and does not diverge, which is crucial to ensure accurate and stable simulations [15]. Due to the dynamic optical properties of the GST, which can be actively tuned by the external electrical and optical signals, the device exhibits an active spectral property by tuning the degree of the crystallization, which can be described as the following formula [16]:

(1) $$\frac{\varepsilon_{eff}\left(p\right)-1}{\varepsilon_{eff}\left(p\right)+2}=p\times \frac{\varepsilon_{c}-1}{\varepsilon_{c}+2}+\left(1-p\right)\times \frac{\varepsilon_{a}-1}{\varepsilon_{a}+2}$$

where $\varepsilon_{c}$ and $\varepsilon_{a}$ represent the relative dielectric constants of crystalline GST (cGST) and amorphous GST (aGST), respectively. The degree of crystallization, p, defines the transition state of the GST material, where p=100% corresponds to fully crystalline GST, and p=0% corresponds to fully amorphous GST. The relative permittivity of the GST at different degrees of crystallization is illustrated in Figure 1c.

The interaction between the mid-infrared light, metasurface, and molecular vibrations can be modeled using two coupled resonators, as illustrated in Figure 1d. The weak absorber **f** typically exhibits minimal interaction with external radiation, whereas the plasmonic resonance *p* strongly couples with incident light. This coupling effectively drives the weak absorber, resembling the interaction between bright and dark modes. Here, the plasmonic mode and the vibrational modes of target molecules act as the bright mode and dark mode, respectively. The coupling between these two modes enables energy transfer from the bright plasmonic mode to the dark molecular mode, effectively “activating” the latter for detection. Specifically, the plasmonic resonance localizes incident light into subwavelength hotspots, amplifying the molecular dipole moment through near-field enhancement. Concurrently, the molecular vibration perturbs the plasmonic resonance, inducing Fano-like spectral distortions (EIT/EIA) that encode the vibrational fingerprint. This coupling mechanism underpins the SEIRA effect, enabling precise reconstruction of molecular spectra. Applying this principle to mid-infrared vibrational modes has significantly advanced surface-enhanced infrared absorption (SEIRA) spectroscopy [17,18,19,20]. In the proposed platform, the SEIRA effect is employed, utilizing the strong near-field enhancement induced by plasmonic resonance to excite the molecular vibrations of the target substance. Consequently, the characteristic fingerprint absorption spectrum of the target molecules can be extracted and reconstructed, achieving the goal of fingerprint spectrum retrieval.

## 3. Results and Discussion

**Figure 2:**
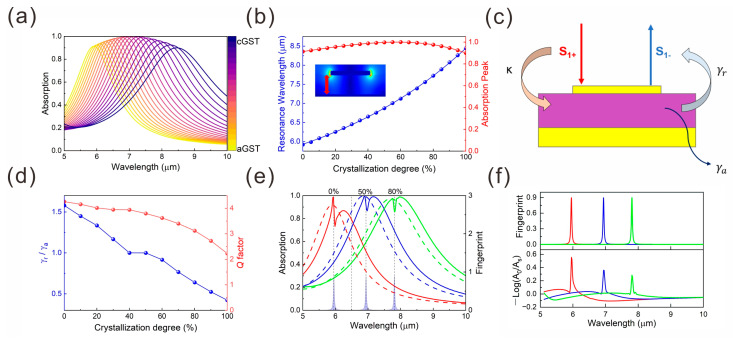
(**a**) Absorption spectra of the device at different crystallization levels of the GST layer. (**b**) The resonant wavelength and absorption peak depend on the crystallization degree. The inset illustrates the electric field distribution at the resonant wavelength when the device operates in the CC state. (**c**) Schematic representation of the time-coupled mode theory (TCMT). (**d**) The ratio of radiation loss to absorption loss and the Q factor of the device for varying crystallization levels of the GST layer. (**e**) Absorption spectra of the device before (dashed line) and after (solid line) loading with a hypothetical test analyte when the device operates at different coupling states. The shaded curve represents the fingerprint of the hypothetical test analytes. (**f**) Reconstructed fingerprint spectra of the hypothetical analytes under different coupling states (**lower panel**), with the fingerprint of the hypothetical analytes shown in the **upper panel**. The red, green, and blue curves in (**e**,**f**) represent the fingerprint spectra reconstructed at crystallinity degree of 0%, 50%, and 80%, respectively.

To evaluate the absorption efficiency of the tunable plasmonic-enhanced infrared absorption platform in the mid-infrared range, we calculated the absorption spectra of the device at various crystallization levels of the dielectric spacer layer, as shown in Figure 2a. As the crystallization degree of GST increases, the spectrum undergoes a redshift, while the maximum absorption intensity remains nearly constant with a value higher than 90%, as depicted in Figure 2b. This behavior is attributed to the excitation of the plasmonic resonance within the structure. The corresponding optical field distribution is shown in the inset of Figure 2b.

We then employed the time-coupled mode theory (TCMT) to analyze the coupling states under various crystallization levels. Taking a single-unit cell structure as an example, as shown in Figure 2c, due to the presence of a reflection metallic plane, only the incident and reflected waves should be considered. Based on TCMT, the coupling equations are expressed as follows [21]:

(2) $$\frac{dP}{dt}=j\omega_{0}P-\left(\gamma_{r}+\gamma_{a}\right)P+\kappa S_{1+}$$

(3) $$S_{1-}=-S_{1+}+\kappa P$$

(4) $$A=1-R=1-{\left|r\right|}^{2}=1-{\left|\frac{S_{1-}}{S_{1+}}\right|}^{2}$$

where *P* represents the modal amplitude of the structure, and $\omega_{0}$ denotes the resonance frequency of the structure. $\gamma_{r}$ and $\gamma_{a}$ correspond to the radiation loss and absorption loss of the structure, respectively. $S_{1+}$ and $S_{1-}$ represent the incident and reflected waves of the structure, while κ is the coupling strength coefficient between the structure and the incident light ($\kappa =\sqrt{2\gamma_{r}}$). Due to the presence of the gold film, the transmitted light spectrum T = 0, simplifying the absorption spectrum to A=1−R. By applying d/dt=jω to Equation (2) and substituting it into Equation (3), the absorption spectrum *A* can be expressed as follows:

(5) $$A=1-{\left|\frac{\kappa^{2}}{j\left(\omega -\omega_{0}\right)+\left(\gamma_{r}+\gamma_{a}\right)}-1\right|}^{2}=\frac{4\gamma_{r}\gamma_{a}}{{\left(\omega -\omega_{0}\right)}^{2}+{\left(\gamma_{r}+\gamma_{a}\right)}^{2}}$$

By fitting Equation (5) to the absorption spectra, the radiation loss $\gamma_{r}$ and absorption loss $\gamma_{a}$ can be extracted and shown in Figure 2d. The results indicate that as the crystallization degree of the GST varies from 0% to 100%, the device can operate at three distinct coupling states: (1) when $\gamma_{r}/\gamma_{a}$ < 1, indicating that the device operates in the under-coupled state (UC state); (2) when $\gamma_{r}/\gamma_{a}$ = 1, placing the device in the critically coupled state (CC state), also referred to as a perfect absorber; (3) when $\gamma_{r}/\gamma_{a}$ > 1, the device operates in the over-coupled state (OC state). The system’s coupling state transitions from OC to CC and finally to UC state as the crystallization level changes. By appropriately adjusting the radiation loss and absorption loss to balance, various excellent micro- and nano- devices based on the perfect absorber can be realized, such as the refractive index sensing and nanometer optical coatings used as labeling and optical filters [22,23,24]. Therefore, this allows for the adjustment of the resonant wavelength without the need to modify the structural parameters of the platform and realize various analytes recognition over a large wavelength range. In the subsequent section, we will explore the platform’s capability to reconstruct molecular fingerprint spectra under these different coupling states.

Numerical simulations were performed for the fingerprint reconstruction when the device operates at three coupling states, as illustrated in Figure 2e. Three distinct absorption peaks are selected at 5.95 µm, 6.94 µm, and 7.81 µm, corresponding to the over-coupled (0% crystallization, aGST), critically coupled state (50% crystallization), and under-coupled (80% crystallization) state, respectively. A 0.3 μm-thick layer of test molecules was placed on the surface of the device. It is evident that the introduction of the analyte induces a significant change in the spectrum. This observation highlights the structure’s high sensitivity to external analytes. For the green solid line, the platform is in the under-coupled (UC) state. Compared to the original absorption spectrum without the test molecule (green dashed line), the spectral distortion is attributed to the electromagnetically induced transparency (EIT) effect; the introduction of the analyte resulted in a decrease in the spectral absorption rate at the central position. When the resonant wavelength of the test molecule aligns with the resonant wavelength of the device operating in the UC state, an interference-like cancelation effect occurs, resulting in reduced absorption at the resonant wavelength. When the platform is in the over-coupled (OC) state, the spectral distortion is caused by the electromagnetically induced absorption (EIA) effect. When the resonant wavelength of the test molecule coincides with the resonant wavelength of the platform in the OC state, an interference-like constructive effect occurs, leading to enhanced absorption at the resonant wavelength. When the device is in the critically coupled (CC) state and the resonant wavelength of the test molecule matches the platform’s resonant wavelength, the absorption spectrum exhibits a similar EIA effect as observed in the OC state. Finally, by comparing the two spectra when the analytes are introduced using the formula $-\log A_{0}/A_{s}$ ($A_{s}\;/A_{0}$ represents the absorption spectrum after/before the analytes have been introduced), the reconstructed spectrum can be obtained, as shown in Figure 2f, demonstrating exceptional reconstruction accuracy.

**Figure 3:**
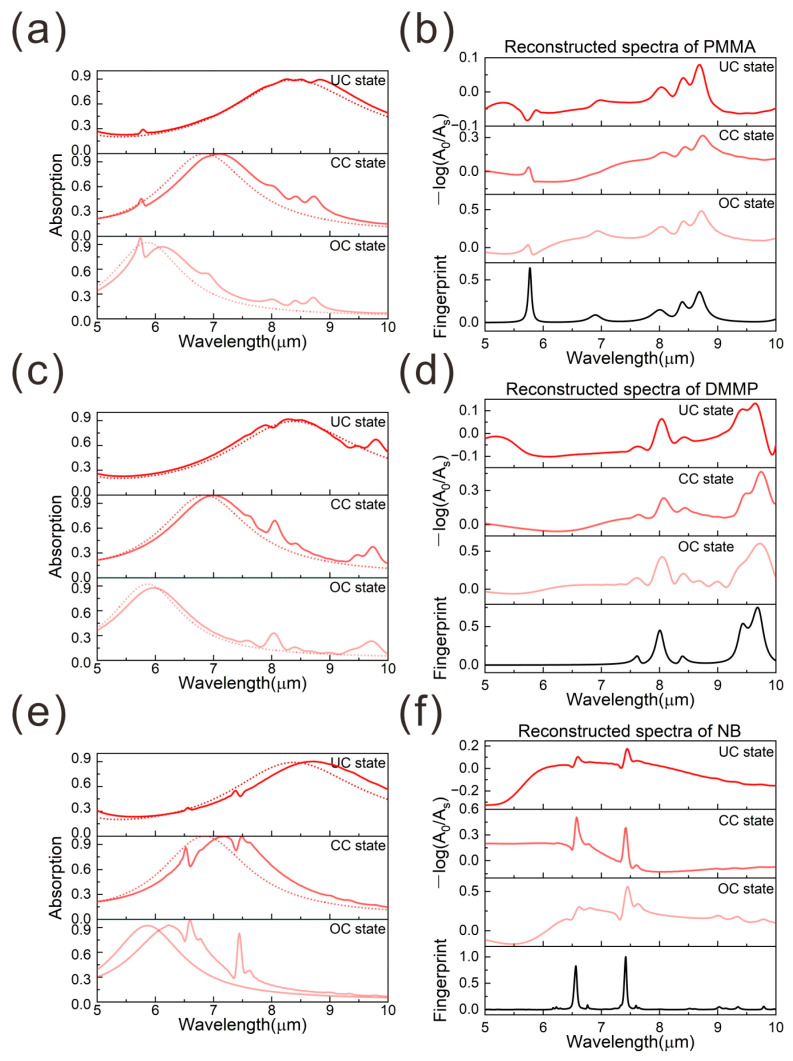
(**a**,**c**,**e**) The absorption spectra of the device loaded with different analytes under three coupling states (solid/dashed lines: without/with the analytes covered). (**b**,**d**,**f**) The reconstructed fingerprint absorption spectra of PMMA, DMMP, and NB molecules under different coupling states. The black curves represent the intrinsic fingerprint of PMMA, DMMP, and NB molecules. The red colors changes from light to dark represent the molecular fingerprint spectra reconstructed by the platform operating at UC, CC, and OC states, respectively.

Based on the excellent reconstruction capacity of the fingerprint either in either OC, CC, or UC states, we implement this method to reconstruct fingerprint spectra of real analytes with complex, multiple characteristic peaks, as well as molecular mixtures. As shown in Figure 3, we investigate the device’s ability to detect and reconstruct the fingerprint spectra of three molecules, including polymethyl methacrylate (PMMA), dimethyl methylphosphonate (DMMP), and nitrobenzene (NB), respectively. The dielectric constant of these analytes is extracted in the previous studies [25,26]. Three coupling states are used to study the reconstruction quality. As discussed above, the introduction of the PMMA with a thickness of 0.3 μm can induce the obvious change in the absorption spectra, as shown in Figure 3a, where the dashed (solid) lines represent the platform’s absorption spectra before (after) loading the analytes. It can be observed that the platform’s resonance mode couples with the vibrational absorption modes of PMMA, causing the measured platform absorption spectra to incorporate the unique fingerprint absorption features of the analytes. The reconstructed spectra under three coupling states are shown in Figure 3b. Note that the prerequisite for successful construction of the fingerprint is the complete coverage of the top surface. The optimal condition for reconstructing the PMMA fingerprint occurs at the OC state, where the plasmonic resonance aligns well with the fingerprint of PMMA, and the interaction between the sensor and the analytes can be strongly enhanced. Similarly, as illustrated in Figure 3c–f, the excellent reconstruction capability for DMMP and NB is recorded at the UC state and CC state, respectively, which further validate the results discussed in fingerprint retrieval of PMMA. Therefore, for sensing different types of analytes, the coupling state of the sensors is different when the sensor exhibits optimal reconstruction capability. In our design, the use of a dynamically tunable phase-change material mitigates this challenge, since the resonance wavelength of the sensor can be easily adjusted without significant fluctuations in the maximum absorption rate. This enables the active tuning of the sensor’s resonance wavelength to align with the primary resonance peaks of target molecules. The effectiveness of coupling states (UC, CC, OC) in fingerprint reconstruction hinges on their spectral alignment with molecular vibrations. For the PMMA fingerprint retrieval, the C = O stretch mode at 1730 cm^−1^ aligns with the plasmonic mode of the sensor when it operates in the OC state. In contrast, for DMMP and NB detection, the P = O stretch mode at 1030 cm^−1^ and aromatic ring modes at 1300–1500 cm^−1^ match the plasmonic mode of the sensor operating in the UC state and CC states, which strongly enhances the light–molecule interaction and enables the fingerprint retrieval. Therefore, when an active material, GST, is integrated onto the plasmonic structure, the dynamic resonances realized in this single device can expand its dynamic range in sensing applications. Consequently, the proposed plasmonic-enhanced infrared absorption platform facilitates molecular fingerprint reconstruction and the distinction of specific molecular species. 

**Figure 4:**
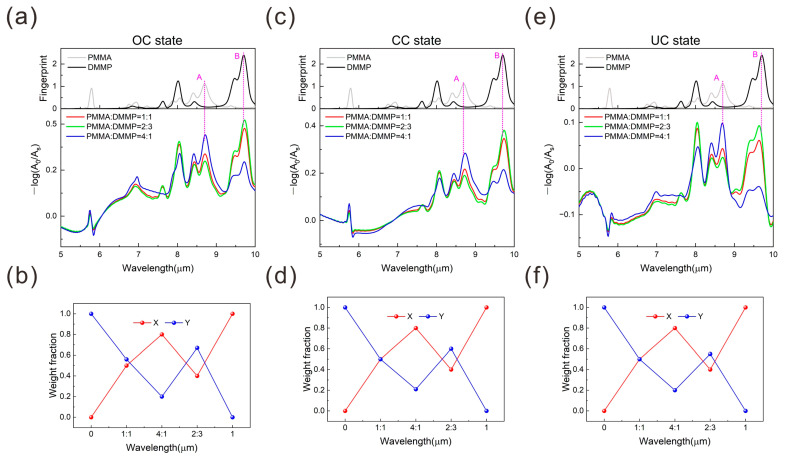
(**a**,**c**,**e**) Reconstructed absorption spectra for PMMA/ DMMP mixtures when the device operates at the OC, CC, and UC state. The red, green, and blue curves correspond to the ratios of 1:1, 2:3, and 4:1, respectively. The peak A and B represent the typical C-O vibration on ester bond structure at 1148 cm ^−1^ and the P = O stretch mode at 1030 cm^−1^ in PMMA and DMMP. (**b**,**d**,**f**) Linear decomposition analysis of the absorption spectra of PMMA/DMMP mixtures with the device operating at the OC, CC, and UC states.

To evaluate the platform’s capability to distinguish the mixed systems containing multiple test substances, we calculated the absorption spectra of PMMA and DMMP molecules at various mixing ratios. The mixtures were prepared with PMMA and DMMP at ratios of 1:1, 2:3, and 4:1, respectively. The complex dielectric constant of the mixture was calculated using the following formula:

(6) $$\varepsilon_{Mixed}={X\varepsilon }_{PMMA}+{Y\varepsilon }_{DMMP}$$

where *X* and *Y* represent the weight fraction of PMMA and DMMP molecules in the mixture, and $\varepsilon_{PMMA}$ and $\varepsilon_{DMMP}$ denote the fingerprint of PMMA and DMMP molecules, respectively. To determine the composition ratios of different molecules in the mixture, a 0.3 μm-thick molecular layer was applied to the surface of the platform for numerical simulations. Figure 4a shows the reconstructed fingerprint spectra of the platform loaded with mixtures at varying ratios when the device operates at OC state (lower panel), along with the fingerprints of PMMA and DMMP molecules in the upper panel. It is evident that as the proportion of PMMA in the mixture increases, the intensity of PMMA’s characteristic fingerprint absorption peaks increases correspondingly, while the intensity of DMMP’s characteristic fingerprint absorption peaks decreases. These variations enable the composition identifications of the mixture. The recognition results for different weight fractions are shown in Figure 4b, indicating the accuracy of the sensor on the composition identification in the mixtures. Similar results are shown in Figure 4c–f when the device operates in the CC state UC state. However, it is noted that the prerequisite for determining the composition in the mixtures is that the intrinsic peak of each analyte can be reconstructed and distinguished, as discussed in Figure 3. Here, the intrinsic peak used for the composition identification is peak A for PMMA and peak B for DMMP, which are labeled in Figure 4a,c,e. Here, the intrinsic peak used for the composition identification is peak A for PMMA and peak B for DMMP, which are labeled in Figure 4a,c,e. However, it should be noted that for some complex systems, such as different molecules with different affinities/steric conformations in proximity to the surface, this simple linear decomposition method will not be sufficient to extract component information. Therefore, sophisticated algorithms and machine learning techniques are required for the identification and component extraction of more complex systems.

## 4. Conclusions

In summary, we present a broadband multi-molecular fingerprint retrieval platform based on plasmonic-enhanced infrared absorption. This platform offers a straightforward structural design and dynamically tunable resonant wavelength, which can actively tune the resonant wavelength to the vibrational fingerprint of the analytes, realizing high-sensitivity fingerprint retrieval. The tunable spectral response is performed by changing the crystallization degree of GST. We show that by changing the crystallization fraction of the GST, the resonant peak can be actively shifted to a broad range, with the absorption peak almost unchanged. Furthermore, we analyze the reconstruction capability of the device when it operates at OC, CC, and UC state, which shows excellent reconstruction when the vibrational fingerprint of the analytes aligns with the resonant wavelength well. Finally, multifingerprint detection with the components distinguished in the mixtures is showcased in the proposed device. Thus, the proposed compact device with reconfigurable properties holds great potential to enable miniaturized, highly sensitive molecular fingerprint detection.

## Data Availability

The data that support the findings of this study are available from the corresponding author upon reasonable request.
